# COVID-19 and what pediatric rheumatologists should know: a review from a highly affected country

**DOI:** 10.1186/s12969-020-00422-z

**Published:** 2020-04-22

**Authors:** Francesco Licciardi, Teresa Giani, Letizia Baldini, Ennio Giulio Favalli, Roberto Caporali, Rolando Cimaz

**Affiliations:** 1 Division of Pediatric Immunology and Rheumatology, Department of Public Health and Pediatrics, Regina Margherita Children Hospital, University of Turin, Turin, Italy; 2 AOU Meyer, V.le Pieraccini 24, 50139 Florence, Italy; 3 Department of Medical Biotechnology, University of Siena, Siena, Italy; 4 Division of Clinical Rheumatology, ASST Gaetano Pini-CTO Institute, Milan, Italy; 5 Department of Clinical Sciences and Community Health, Research Center for Adult and Pediatric Rheumatic Diseases, Università degli Studi di Milano, Milan, Italy

**Keywords:** 2019-nCoV, SARS-CoV-2, COVID-19, Children, Respiratory syndrome, Italy

## Abstract

On March 11th, 2020 the World Health Organization declared COVID-19 a global pandemic. The infection, transmitted by 2019 novel coronavirus (2019-nCov), was first discovered in December 2019, in Wuhan, Hubei Province, and then rapidly spread worldwide. Italy was early and severely involved, with a critical spread of the infection and a very high number of victims. Person-to-person spread mainly occurs via respiratory droplets and contact. The median incubation period is 5 days. The spectrum of respiratory symptoms may range from mild to severe, strictly depending on the age of the patient and the underlying comorbidities.

In children COVID-19 related disease is less frequent and less aggressive. In Italy 1% of positive cases are under 18 years of age, and no deaths have been recorded before 29 years of age. For patients affected by rheumatic disease, despite the concerns related to the imbalance of their immune response and the effect of immunosuppressive treatments, there are still few data to understand the real consequences of this infection. Major scientific societies have issued recommendations to help rheumatologists in caring their patients. Interestingly, some of the drugs mostly used by rheumatologists appear to be promising in critical COVID-19 infected patients, where the hyperinflammation and cytokine storm seem to drive to the multiorgan failure.

Pediatric rheumatologists are expected to play a supporting role in this new front of *COVID*-19 pandemic, both as general pediatricians treating infected children, and as rheumatologists taking care of their rheumatic patients, as well as offering their experience in the possible alternative use of immunomodulatory drugs.

## Background

In December 2019, an outbreak of pneumonia of unknown etiology occurred in Wuhan, Hubei province of China. On January 7th, the Chinese Center for Disease Control and Prevention (CDC) isolated a novel coronavirus from the throat swab sample of a patient and identified it as the etiologic agent of this cluster of pneumonia; the World Health Organization (WHO) named the virus 2019-nCov and COVID-19 the related disease. Then, the Coronavirus Study Group of the International Committee on Taxonomy of Viruses proposed the new virus to be designated severe acute respiratory syndrome coronavirus 2 (SARS-CoV-2) [1].

## Epidemiology

Since the first reports of cases from Wuhan, SARS-CoV-2 outbreak followed an exponential growth [2] and rapidly spread across China, leading the WHO Emergency Committee to declare a global health emergency on January 30th, 2020. Then the epidemic extended to many other countries across all continents so that COVID-19 was recognized as a pandemic by the World Health Organization (WHO) on March 11th, 2020.

Global cases as of March 23rd have now exceeded 330,000. After China, Italy is the country most affected by the spread of COVID-19 infection. Italian epidemiologic data updated to March 24th, show a very critical situation with a total of 69,176 confirmed infected people, of which 8326 have recovered and 6820 are deceased [3]. Therefore, at this time this is written, Italy is the nation with the most deaths due to COVID-19 worldwide; the reason of this may be related to different factors such as timing and modalities of confinement, older median age, rate of smoking and pollution. Moreover, reported deaths in our system include all cases deceased *with* coronavirus rather than *for* coronavirus; clinical chart review is ongoing to detect causality. The lack of the precise number of asymptomatic subjects also prevents the possibility to calculate the true infection fatality rate, that strictly depends on the extent of the swab test to the whole general population. While asymptomatic infections have in fact been described, their exact prevalence is still unknown [4]. In order to estimate the proportion of asymptomatic subjects all 3711 people on board of the Diamond Princess cruise ship were tested, and the percentage of asymptomatic subjects was 17.9% (95% CI 15.5–20.2%) [5].

## Viral characteristics and transmission

Coronaviruses are enveloped, non-segmented positive-sense RNA viruses whose name is due to club-shaped spike projections emanating from the surface of the virion, which give them the appearance of a solar corona. Virions structure is composed of the spike (S), membrane (M), envelope (E) and nucleocapsid (N) proteins. Sars-CoV-2 gains entrance in lung cells thanks to the interaction between the S protein and angiotensin-converting enzyme 2 (ACE2) [6, 7]. Following replication and sub-genomic RNA synthesis, the viral structural proteins, S, E, and M are inserted into the endoplasmic reticulum (ER), where viral genomes forms mature virions, that are transported to the cell surface in vesicles and released by exocytosis [8].

Coronaviruses can be divided into four genera: alpha, beta, delta, and gamma. Before SARS-CoV-2 outbreak, six coronaviruses -belonging either to alpha or to beta group- were known to cause respiratory diseases in humans: four of them — 229E, OC43, NL63, and HKU1 —typically provoke upper respiratory tract, while two other strains — severe acute respiratory syndrome coronavirus (SARS-CoV) and Middle East respiratory syndrome coronavirus (MERS-CoV) —are responsible for severe human pneumonia. Full-genome sequencing revealed that Sars-CoV-2 belongs to betacoronavirus but diverges from MERS-CoV and SARS-CoV [6, 9]. Considering SARS-Cov-2 genetic similarity to RaTG13 bat coronavirus, with whom it shares 96% of the genome, the hypothesis that 2019-nCoV has originated from bats is likely [10]. Both SARS-CoV and MERS-CoV originated in bats and were transmitted to human by an intermediate host [11]; in analogy, some authors suggest that pangolin, mink, snake or turtle may be potential intermediate hosts for the virus [12–18].

At first, the vast majority of affected patients were linked to the Wuhan Seafood Market suggesting possible animal and environmental exposures*.* Soon, however, person-to-person spread was reported [4] and became the main mode of transmission. Both asymptomatic and pre-symptomatic patients can transmit the infection [19, 20] and the spread seems to occur mainly via droplets. Some authors detected peak concentration of the virus in upper airways before day 5 from the onset, with the shedding of viral RNA from sputum outlasting the end of symptoms [20]. As far as clinical impact of SARS-CoV-2 infection on pregnancy is concerned, currently we have very limited knowledge, but there is currently no evidence of vertical transmission during the third trimester [21].

## Clinical features

COVID-19 manifests itself after a median incubation period of about 5 days (95% CI, 4.5 to 5.8 days), with a range of 0–24 days and the vast majority (97.5%) of patients becoming symptomatic 11.5 days (CI, 8.2 to 15.6 days) from infection [22, 23]. Classical flu-like symptoms initially include fever, dry cough, fatigue, rhinorrhea, myalgia, and less frequently headache and diarrhea. The infection can progress, affecting the lower respiratory tract inducing dyspnea, increased respiratory frequency, decreased oxygen saturation, until respiratory failure, septic shock, and multiorgan dysfunction [9, 22, 24, 25].

Many patients seem to report loss of smell and taste during or after SARS-CoV-2 infection. Imaging studies show that more than 75% of patients have bilateral lung involvement [9, 24, 25], and multilobe involvement is also common. Multifocal patchy bilateral ground glass opacities, consolidation, and bronchiectasis are the typical abnormalities observed on computed tomography (CT) of the chest, with a preferential peripheral distribution, and lower lung involvement [26, 27].

Laboratory data usually show lymphopenia, decreased albumin, high values of C-reactive protein (CRP), erythrocyte sedimentation rate (ESR), and lactate dehydrogenase [28]. Significantly high circulating levels of cytokines and chemokines were noted in patients with COVID-19 infection, with plasma concentrations of IL-2, IL-7, IL-10, G-CSF, IP-10, MCP-1, MIP-1A, and TNF-α being higher in ICU patients than non-ICU patients [9]. Higher levels of neutrophils, AST, LDH and CRP and lower level of platelets and albumin are described in refractory patients [29], while old age, presence of underlying diseases, and elevated inflammatory markers seem to be predictors of a fatal outcome [30].

## COVID-19 in children

Regarding the pediatric age, it is now known that COVID-19 related disease is less frequent and less aggressive. In Italy only about 1% of positive cases are under 18 years of age, and no deaths have been recorded before 29 years of age [3]. Numbers collected by the Chinese Center for Disease Control and Prevention reveal that children age 1–10 years old represent only 0.9% of COVID-19 cases while children and adolescents age 10–19 years old were 1.2% of the 44,672 confirmed positive subjects.

Dong et al. studied the epidemiologic characteristics of 2143 pediatric patients with suspected or confirmed infection identified from January 16th to February 8th 2020 in China [31]. The authors described an asymptomatic, mild, or moderate course in 94.1% of cases, with 4.4% of patients totally asymptomatic. They considered asymptomatic those children with positive laboratory tests without any clinical signs or symptoms or radiological chest findings, mild those with symptoms of acute upper respiratory tract infection in the absence of auscultatory abnormalities or children with only digestive symptoms, and moderate those with pneumonia, but no obvious hypoxemia. The spectrum of manifestations observed in the pediatric age includes fever (frequently low grade), cough, pharyngeal erythema, tachycardia, and tachypnea, less usually rhinorrhea, diarrhea, vomiting, and fatigue [31]. Children may show a coinfection with other viral pneumonia, and in a series of 20 Chinese children eight showed a concomitant infection with influenza viruses A and B, respiratory syncytial virus, Mycoplasma pneumoniae or cytomegalovirus [32]. The majority of pediatric patients have a normal blood count, with 15% showing leukocytosis and 15% leukopenia. CRP and ESR are also frequently in normal range [33]. Hence, while in adults lymphocyte and platelet counts, CRP, and albumin levels have been proposed as signs for severe infection, in children the potential prognostic value of these indicators is not clear [33]. Chest CT findings are documented in almost half of children, and appear to be similar, but milder when compared to those found in adults [33, 34]. Plain chest X-rays may fail to detect the lesions or may be unable the detail pathologic features [32].

Finally, the experience on neonates and mother-child transplacental transmission is very limited. Pregnant women can have the typical features of COVID-19 pneumonia present in non-pregnant women, but outcomes in their neonates seem to be favorable [35]. Data obtained from a small group of women with COVID-19 pneumonia in their third trimester of pregnancy do not support an intrauterine infection caused by vertical transmission [21].

## Juvenile idiopathic arthritis and risk of infections

Patients with juvenile idiopathic arthritis are more prone to infections, especially during periods of disease activity. On top of that, anti-rheumatic drugs may increase this susceptibility, and serious infections are the most common adverse events that might be related to biologic agents [36]. However, data from large epidemiological studies as well as national registries have been more reassuring than once hypothesized [37, 38]. For example, the rate of hospitalized bacterial infections among patients diagnosed with JIA and with attention deficit hyperactivity disorder showed that the spectrum of infections was comparable in both groups.

An increased rate of infections was found in JIA patients treated with more than 10 mg/day of prednisone. The use of immunosuppressants and the risk of infections has been studied with particular attention on TNF inhibitors. However, definitions and cohorts differed among the published articles. In a study coming from the British Society for Paediatric and Adolescent Rheumatology [39] an increased incidence of medically significant infections among patients treated with etanercept compared to methotrexate (MTX) users was found (aHR 2.13, 95% CI 1.22–3.74). The German Biologic Registry for Pediatric Rheumatology found an increased risk of severe infections either for etanercept and adalimumab compared to MTX (hazard ratio of 6.0, 95% CI 2.0–17.5, and 7.3, 95% CI 1.3–40.0, respectively), with no difference between etanercept and adalimumab. We know that disease activity can be a predisposing risk for infections, and indeed in this study the clinical Juvenile Arthritis Disease Activity Score was found to be an independent risk factor for infections in multivariate analysis [40]. Lee et al. showed concerns on the risk of infections in JIA patients receiving TNF inhibitors [41]. They identified an increased risk of bacterial infections requiring hospitalization compared to DMARDs users, with an adjusted hazard ratio of 2.72 (95% CI: 1.08–6.86); however, they did not consider the possible influence of previous MTX treatment.

Of note, and relevant to the COVID-19 epidemic, in the aforementioned studies the respiratory system was usually the preferred site of infections. With regard specifically to viral infections, influenza has been quite extensively studied, also for the possibility of seasonal vaccination [42]. Among others, the incidence of Herpes Zoster Virus infections has been found to be almost three-fold higher in JIA patients [43]. Also, despite early vaccination the possibility of hepatitis B virus reactivation during anti-TNF treatment has been reported [44]. The incidence of COVID-19 among patients with JIA is yet unknown. Our own experience so far is too short to make conclusions or statements, but up to now we are not aware of any in our large cohorts.

## Treatment of COVID-19 and possible role of anti-rheumatic drugs

Currently there is no available vaccine for COVID-19 and treatment is mainly supportive. Many different therapies are now under investigation in order to define as soon as possible a targeted therapy to treat the infection and reduce overall mortality. Different anti-viral therapies have been tested but strong evidence of efficacy is so far lacking. Early reports have described a possible benefit of lopinavir-ritonavir therapy [45]; therefore this molecule is now included in severe case management protocols [46]. Unfortunately a recently published RCT on 199 adult patients with severe disease has not confirmed previous preliminary results, demonstrating similar mortality and time to clinical improvement in treatment and placebo arms [47]. The role in milder cases is still debated.

Remdesivir is an adenosine analogue originally developed for the treatment of Ebola. It efficiently inhibits coronavirus infection of human cells in vitro [48] therefore it may be useful in vivo. Although the drug has not been approved by FDA and EMA, two Phase III trials are ongoing in the USA in severe (NCT04292899) and moderate (NCT04292730) COVID-19 infections. Preliminary results are not available yet.

While evidence of anti-viral benefit in vivo is still lacking recent reports have suggested that drugs commonly used in rheumatology settings may have an important role in COVID-19 management.

### Chloroquine/hydroxychloroquine

Chloroquine (CQ) and hydroxychloroquine (HCQ) have been used as anti-malarial drugs for decades, furthermore thanks to its immunomodulatory properties HCQ is currently used for the management of autoimmune diseases such as SLE and RA [49]. Studies have demonstrated that both drugs in vitro are able to interfere with different viruses [50, 51]. In vitro CQ decreases the glycosylation of ACE2 hindering the entrance of the virus in the host cells. Furthermore, CQ and HCQ increase the pH of endosomes in cells preventing the fusion process between host and viral membrane [52]. HCQ therapy may be useful in COVID-19 infections not only for its anti-viral activity; the ability to decrease cytokine production, suppress TLR signaling, and decrease interferon pathway activation may dampen the inflammatory phase of the disease [52]. Finally, HCQ has also proven some efficacy in preventing recurrent acute respiratory distress syndrome in children with interstitial lung disease due to monogenic surfactant deficiency [53]. At least 16 different trials are ongoing in China, and six others are registered, for testing the efficacy of CQ and HCQ in the treatment of COVID-19. Preliminary results by Gautret et al. suggest that HCQ 600 mg daily may decrease viral load in nasal swabs [54]; furthermore, Gao reports that “chloroquine phosphate is superior to the control treatment in inhibiting the exacerbation of pneumonia, improving lung imaging findings, promoting a virus negative conversion, and shortening the disease course” [55]. In a recent report Yao et al. have demonstrated in a pharmacokinetic model in vitro that HCQ is 3-times more potent than CQ; according to this study a loading dose of 400 mg twice daily of HCQ given orally, followed by a maintenance dose of 200 mg given twice daily for 4 days is the therapeutic regimen recommended [56].

### IL-6 and IL-1 blockers

Critically ill patients with COVID-19 admitted to intensive care unit (ICU) care or with fatal outcome have significantly higher cytokine levels when compared to patients with milder disease, suggesting that cytokine storm may play a role in COVID 19 mortality [9, 57]. In particular, Zhou et al. have demonstrated in a retrospective study that survivors had significantly lower IL-6 levels than non-survivors [57]. Preliminary results of Tocilizumab (TCZ) use in COVID-19 have been provided by Xu et al., who reported a marked improvement in 21 patients treated with TCZ in terms of decreased O_2_ need, decreased inflammatory markers and resolution of CT lesions [58]. Based upon these findings a single arm phase 2 study has also been approved by the Italian Regulatory Drug Agency and will enroll patients with pneumonia and early respiratory failure, with mortality reduction at 1 month as primary outcome (EudraCT number 2020–001110-38). Participants will receive 1 dose of TCZ (8 mg/Kg), and a second dose could be given after 12 h if respiratory function has not recovered. In the U.S. another phase 2 trial with the IL-6 blocker Sarilumab is currently ongoing (NCT04315298).

The importance of cytokine storm in COVID-19 pneumonia may suggest that other cytokine blockers may be beneficial in the treatment of severe cases. Interestingly, biomarkers of disease progression in COVID-19 resembles well known markers of macrophage activation syndrome such as high ferritin, low platelet, high ALT [9, 57]; considering these similarities treatment with IL-1 blockers may be beneficial. A phase 2/3, randomized, open-label multicenter study investigating the efficacy and safety of emapalumab (anti-IFNγ) and anakinra versus standard of care in reducing hyper-inflammation and respiratory distress in patients with SARS-CoV-2 infection is currently ongoing (EudraCT Number: 2020-001167-93) [59].

### NSAID safety

On March 14th, 2020 France Health authorities delivered a warning regarding the use of NSAIDs in patients affected by COVID-19 due to possible severe side effects [60]. On March 18th, EMA declared that there is currently no scientific evidence establishing a link between ibuprofen and worsening of COVID-19. EMA recommends “When starting treatment for fever or pain in COVID-19, patients and health care professionals should consider all available treatment options including paracetamol and NSAIDs. Each medicine has its own benefits and risks which are reflected in its product information and which should be considered along with EU national treatment guidelines, most of which recommend paracetamol as a first treatment option for fever or pain.” While evidence regarding possible side effects of NSAID is lacking, it has been suggested that Indomethacin, an NSAID used in systemic JIA, has a potent antiviral activity against coronavirus [61].

## Practical points for pediatric rheumatologists

Pediatric rheumatologists are now daily confronted with patients and families requesting informations on how to proceed with regard to scheduled appointments, infusion visits, as well as asking whether any change of therapy should be advised due to possible increased risk of susceptibility to infection secondary to immunosuppressive treatments (in particular corticosteroids and biologics). Scientific societies such as the Italian Society of Rheumatology, the Pediatric Rheumatology European Society and the European League against Rheumatism have issued recommendations, easily downloadable from their web sites. We obviously follow these statements, as well as advise families to follow the individual country Ministry of Health/National public health care body’s recommendations. Our adult rheumatology department has issued relevant statements related to COVID-19 and rheumatoid arthritis [62].

In our centers we always underline the more simple measures to prevent contacts with infected subjects and spread of virus, i.e. often cleaning hands with soap or alcohol-based sanitizers, avoiding contacts with people who manifest respiratory symptoms, keeping the safe social distance of at least one meter, coughing or sneezing not on palms but into elbow, using disposable tissues and tell patients and families to avoid touching their face as much as possible. Emergency department visits have to be avoided unless strictly necessary, but the primary care physician or pediatrician should be contacted in case of fever or respiratory symptoms. Outpatient consultations and elective hospitalizations should be deferred. An on-call number to answer specific questions has been created in our center and is very useful especially for patients who miss their appointments.

At the moment, recommendations of rheumatologic societies both in Italy and Europe suggest to continue all immunosuppressant therapies as usual, since medications withdrawal may cause a flare of inflammatory disease, which in turn can lead to higher infectious risk. With regard to drug delivery for hospital-prescribed medications, several prescriptions in Italy have been automatically renewed for 90 days. At the present time swabs are not prescribed for asymptomatic individuals, but screening on health care providers is discussed. These (ie ourselves) are the next most important people to protect, since everyday contact is allowed by definition for physicians and asymptomatic people could theoretically carry the virus and potentially be a contagious source, with dangerous spread not only for children but for family contacts and in particular elderly subjects who are at risk for severe course of disease. Therefore use of personal protective equipment depending on specific clinical situations is required.

## Conclusion

The COVID-19 epidemic is now a pandemic and may affect millions of people worldwide. For the time being, children seem to be spared, at least from the more severe consequences of this infection. All physicians dealing with patients with chronic diseases, in particular immunosuppressed subjects, should be aware of the possible risks linked to the drugs used to treat rheumatologic disorders. However, there now hints that some of these drugs might be beneficial to fight COVID-19 infection. Use of social isolation and hygienic measure are fundamental in order to decrease viral spread.

## Data Availability

Not required (Data sharing is not applicable to this article as no data are included, nor datasets were generated or analyzed).

## Abbreviations

CDC: Center for Disease Control and Prevention
WHO: World Health Organization
SARS-CoV-2: Acute respiratory syndrome coronavirus 2
S: Spike
M: Membrane
E: Envelope
N: Nucleocapsid proteins
ACE2: Angiotensin-converting enzyme 2
ER: Endoplasmic reticulum
SARS-CoV: Severe acute respiratory syndrome coronavirus
MERS-CoV: Middle East respiratory syndrome coronavirus
CT: Computed tomography
CRP: C-reactive protein
ESR: Erythrocyte sedimentation rate
LDH: Lactate dehydrogenase
JIA: Juvenile idiopathic arthritis
CQ: Chloroquine
HCQ: Hydroxychloroquine
TCZ: Tocilizumab
